# The approach of Norm Balance in predicting pharmacists’ intention to collaborate with physicians to improve medication therapy

**DOI:** 10.3389/fphar.2024.1375529

**Published:** 2024-09-16

**Authors:** Yifei Liu, Karen B. Farris, Dhananjay Nayakankuppam, Bernard A. Sorofman, Julie M. Urmie, William R. Doucette

**Affiliations:** ^1^ Division of Pharmacy Practice and Administration, University of Missouri—Kansas City School of Pharmacy, Kansas City, MO, United States; ^2^ Department of Clinical Pharmacy, College of Pharmacy, University of Michigan, Ann Arbor, MI, United States; ^3^ Department of Marketing, Tippie College of Business, University of Iowa, Iowa City, IA, United States; ^4^ Department of Pharmacy Practice and Science, College of Pharmacy, University of Iowa, Iowa City, IA, United States

**Keywords:** the Theory of Planned Behavior, Norm Balance, subjective norm, self-identity, pharmacist-physician collaboration, medication therapy

## Abstract

**Objective:**

Norm Balance is an approach under the Theory of Planned Behavior (TPB) where subjective norm is weighted by the relative importance of others and self-identity is weighted by the relative importance of self. The relative importance was measured previously by a trade-off measure. In this study, we developed separate measures for the relative importance. The study objectives were to: 1) assess the construct validity of the separate measures; 2) examine the approach of Norm Balance in predicting pharmacists’ intention to collaborate with physicians to improve medication therapy; and 3) establish a modified TPB.

**Methods:**

We selected a random sample of 750 Iowa pharmacists and conducted two surveys. The first survey was to examine intention prediction, and the second survey was to examine behavior prediction by measuring behavior among respondents to the first survey. The relative importance was measured by both the trade-off measure and the separate measures. Exploratory factor analyses were performed for the relative importance of others (separate measures) and subjective norm, and for the relative importance of self (separate measures) and self-identity. Regressions for intention prediction were conducted for TPB with self-identity and the approach of Norm Balance. The same regressions were also conducted for three subgroups according to the median of the relative importance of others (trade-off measure). Moreover, another regression was conducted for behavior prediction.

**Results:**

239 practicing pharmacists responded to the first survey, and 188 responded to the second survey. The separate measures had cross factor loadings, whereas the trade-off measure had low correlations with other constructs. Both regressions for intention prediction explained 75% of the variance, with self-efficacy and attitude being strong predictors. Self-identity was not a predictor in the TPB with self-identify, but self-identity weighted by the relative importance of self was a significant predictor in the approach of Norm Balance. Regression coefficients of subjective norm and self-identify varied across subgroups. The regression for behavior prediction explained 30% of the variance, with intention and self-efficacy being two predictors.

**Conclusion:**

The trade-off measure was better than separate measures. The approach of Norm Balance appears to be a better model than the TPB with self-identity to examine pharmacist-physician collaboration.

## Introduction

Expenditures due to medication-related morbidity and mortality in the U.S. were estimated to be between $495.3 billion and $672.7 billion in 2016 (Watanabe et al., 2018). These costs are likely to increase as the aging population develops chronic conditions that require long-term medication therapy. One healthcare professional alone may not be able to address the issue of medication-related problems. Collaboration between pharmacists and physicians to solve medication therapy problems has been shown to have a positive impact on improving medication therapy in the state of Iowa (Chrischilles et al., 2004). However, there are barriers to pharmacist-physician collaboration. For example, in the state of Missouri, pharmacists were concerned about time and reimbursement for services, and physicians were concerned about the disconnect between healthcare professionals and inadequate pharmacist training (Koval et al., 2021). In addition, the extent of collaboration varies among pharmacists who do have a collaborative relationship (McDonough and Doucette, 2001), and the causes of variation in pharmacist-physician collaboration are not fully understood (McDonough and Doucette, 2001; Doucette et al., 2005). Nevertheless, the challenging times of the COVID-19 pandemic have highlighted the importance of this collaborative effort as never before (Waszyk-Nowaczyk et al., 2021).

The Theory of Planned Behavior (TPB) can be used as a framework to examine pharmacists’ intention to collaborate with physicians to improve medication therapy. In TPB, the determinants of intention are attitude toward the behavior, subjective norm, and perceived behavioral control (Ajzen, 1991). Intention is considered the immediate antecedent of the behavior, attitude toward the behavior refers to the degree to which an individual has a favorable or unfavorable evaluation of the behavior, subjective norm involves the perception of whether important others think the individual should engage in the behavior, and perceived behavioral control reflects the perception of the individual’s ability to perform the behavior. Perceived behavioral control can directly affect both intention and the behavior itself. Self-identity reflects the extent to which individuals perceive themselves as having a role (Stryker, 1968; Stryker et al., 1982) and has been shown to be a useful additional predictor of intention in the TPB (Conner and Armitage, 1998; Sparks and Guthrie, 1998; Armitage and Conner, 1999a; Armitage and Conner, 1999b; Pierro et al., 2003; Hagger and Chatzisarantis, 2006). As both subjective norm and self-identity are normative concepts (Conner and Armitage, 1998), Liu et al. proposed an innovative approach of Norm Balance which places these two constructs under the domain of “norm” (Liu et al., 2023). Subjective norm and self-identity have their own degree of influence and relative importance within this domain. The influence is captured by the measurement score for each construct, and their relative importance indicates how much each construct contributes to the domain of “norm”. Therefore, in this approach, subjective norm and self-identity need two parameters to be fully measured. Each of their measurement scores should be weighted by their relative importance to the individual. The two parameters measuring self-identity and subjective norm would vary independently. Liu et al. also developed a trade-off measure of the relative importance by asking individuals to allocate 10 points between their important others and themselves. Across intentions of six behaviors, they found that the TPB with the approach of Norm Balance explained similar intention variance to the TPB with self-identity, but provided a different perspective on the predictive utility of subjective norm and self-identity (Liu et al., 2023).

Building on the study of Liu et al. (Liu et al., 2023), the present study developed three items to measure the relative importance of others, and another three items to measure the relative importance of self. These items were referred to as the “separate measures” to distinguish from the trade-off measure. The study objectives were to: 1) assess the construct validity of the separate measures for the relative importance of others vs self; 2) examine the approach of Norm Balance in predicting pharmacists’ intention to collaborate with physicians to improve medication therapy; and 3) establish a modified TPB with the approach of Norm Balance. This study was the first to examine pharmacist-physician collaboration using the approach of Norm Balance, and it has both practical and theoretical contributions. First, it contributes to the literature on improving medication therapy by providing insight into pharmacist-physician collaboration. Second, it contributes to measuring the relative importance of others vs self, under the approach of Norm Balance for predicting intention. Third, it contributes to improving predicative performance of the TPB by considering intention prediction, behavior prediction, and the approach of Norm Balance.

## Methods

The instruments of this study were included in two surveys of a longitudinal study that examined pharmacist-physician collaboration (Liu et al., 2010; Liu and Doucette, 2011), so this study shared the same study sample and procedures. However, this was a different study focusing on the approach of Norm Balance under the TPB, while the longitudinal study focused on the Model of Collaborative Working Relationship. The study sample was 750 Iowa pharmacists randomly selected from a sample frame of pharmacists provided by Iowa Pharmacy Association. The sample size was estimated through the formula of simple random sampling, and the estimation method was described in a previous study (Liu et al., 2010). Specifically, the formula is n ≥ z^2^ * (1—P)/(ε^2^ * P), where n is the sample size, z is the reliability coefficient (z = 1.96 for a 95% confidence level), ε is set to make sure that sample estimate d should not differ from the unknown population parameter D by more than ε * D, and P is the unknown population proportion (Levy et al., 1999). P should be the proportion of pharmacists working with physicians to improve medication therapy. One study reported that 72% of Iowa pharmacists intended to provide Medication Therapy Management services (Herbert et al., 2006), and another study implied that 50% of pharmacists agreed that they collaborated with physicians (Doucette et al., 2005). Thus, P was estimated to be 36% (72% * 50%). When ε was set to be 0.15 and z was set to be 1.96, the sample size should be 304. The useable response rate was estimated at 42.2% (Doucette et al., 2005; Herbert et al., 2006), which means 721 subjects need to be contacted (304/42.2%). Therefore, the sample size was set at 750.

Two surveys were employed each using three contacts with subjects: an initial survey packet, a reminder card approximately 2 weeks after the initial survey packet, and another survey packet for non-respondents approximately 4 weeks after the initial survey packet (Dillman, 1999). The first survey included the measures of attitude toward behavior, subjective norm, self-efficacy (representing perceived behavioral control), self-identity, and intention, each measured by three items with a seven-point scale. The second survey included a two-item behavior measure with a seven-point scale for respondents to the first survey. All the measures were modified from reliable and valid instruments (Supplementary Appendix SA) (Armitage and Conner, 1999a; Armitage and Conner, 1999b; Rhodes and Courneya, 2003). The context of measurements was “collaborating with the physician to improve medication therapy in the next 3 months”. This context including the timeframe of 3 months was chosen since pharmacist-physician collaboration would be intended to take place in the next two or 3 months (McDonough and Doucette, 2001). At the beginning of the survey, it was made clear that “the physician” could be one physician with whom the pharmacist was currently working, or one with whom the pharmacist had not worked.

For the approach of Norm Balance, the relative importance of others vs self was measured by both the trade-off measure and the separate measures. The trade-off measure was:

*“Please allocate 10 points between the two sources below to indicate the extent of their impact on your decision to collaborate with the physician to improve medication therapy.*

*People who are important to you____; Yourself____”*.

The trade-off measure was consistent with what was used by Liu et al. (Liu et al., 2023). The separate measures of relative importance of others were developed as the following (“1 = strongly disagree” and “7 = strongly agree”).

(1) *“I highly care about the opinions of people who are important to me on whether I should collaborate with the physician to improve medication therapy.”*
(2) *“I greatly value the opinions of people who are important to me on whether I should collaborate with the physician to improve medication therapy.”*
(3) *“The opinions of people who are important to me have strong influence on my decision to collaborate with the physician to improve medication therapy.”*

Similarly, the separate measures of relative importance of self were developed as the following (“1 = strongly disagree” and “7 = strongly agree”).

(1) *“I highly care about my own opinions on whether I should collaborate with the physician to improve medication therapy.”*
(2) *“I greatly value my own opinions on whether I should collaborate with the physician to improve medication therapy.”*
(3) *“My own opinions have strong influence on my decision to collaborate with the physician to improve medication therapy.”*

A pilot test of the instruments in the first survey was conducted among 150 Iowa pharmacists. The reliabilities of measures in the pilot study were high (Cronbach’s alpha ≥0.88) (Nunnally, 1978), so no changes were made for these measures in the full study.

Measures were calculated for each person by taking the mean of the corresponding items for attitude toward behavior, subjective norm, self-efficacy, self-identity, intention, the relative importance of others (separate measures), the relative importance of self (separate measures), and behavior. Descriptive statistics and bivariate correlations were calculated. Reliability analyses were performed for multiple-item measurements. Cronbach’s alpha was calculated for 3-item construct measures (Nunnally, 1978) and Spearman-Brown Coefficient was calculated for 2-item behavior measure (Eisinga et al., 2013). In addition, to examine the construct validity of the separate measures of the relative importance of others vs self, two exploratory factor analyses using principal axis factoring with oblique rotation were performed (Fabrigar et al., 1999). One factor analysis examined items of the relative importance of others (separate measures) and subjective norm. The other factor analysis examined items of the relative importance of self (separate measures) and self-identity.

Two regressions were conducted for the second objective. The first regression examined the TPB with the addition of self-identity, in which intention was regressed on attitude toward behavior, subjective norm, self-efficacy, and self-identity. The second regression examined the approach of Norm Balance under the TPB, in which intention was regressed on attitude toward behavior, subjective norm weighted by the relative importance of others, self-efficacy, and self-identity weighted by the relative importance of self. In addition, subjects were divided into three subgroups according to the median of the relative importance of others (the trade-off measure), because there were similar numbers of subjects in groups above and below the median, and these two groups were of interest for a clear comparison. The two regressions also were also conducted for each subgroup. Lastly, another regression was conducted for the third objective, in which behavior was regressed on intention and self-efficacy. Collinearity was assessed by tolerance and variance inflation factor (VIF), and usually a VIF greater than 10 indicates collinearity (Kleinbaum et al., 1998). The data analyses were conducted by Statistical Package for the Social Sciences (Chicago: SPSS Inc.). Both the pilot test of survey instruments and the full study were approved by the Institutional Review Boards of the University of Iowa.

## Results

For the first survey, with a response rate of 33%, 239 practicing pharmacists were included in data analyses (Liu et al., 2010; Liu and Doucette, 2011). Their average age was 39.8 years, 60% of them were female, and 98% were Caucasian. For the second survey, 188 out of the 239 pharmacists responded (Liu et al., 2010). However, not all respondents completed all items or questions in each survey. All multi-item measures had reliability ≥0.73 (Table 1). The average measurement scores for intention, attitude, subjective norm, self-efficacy, and self-identity were at least 5.31, while the average measurement score for behavior was 4.25. Both the trade-off and separate measures of relative importance of others vs self, indicated that subjects valued their own opinions about collaboration more than others. Table 2 shows the frequency of the trade-off measure for the relative importance of others vs self. The median for the relative importance of others was 4.

**TABLE 1:** Reliability, mean, and standard deviation of construct measures.

Construct	Reliability^a^ (N)	Means (SD)
TPB constructs and self-identity^b^		
Intention (3 items)	0.91 (237)	5.45 (±1.41)
Attitude (3 items)	0.88 (236)	6.20 (±0.85)
Subjective norm (3 items)	0.73 (237)	5.31 (±1.10)
Self-efficacy (3 items)	0.92 (236)	5.43 (±1.48)
Self-identity (3 items)	0.90 (239)	5.57 (±1.24)
Behavior (2 items)	0.82 (188)	4.25 (±1.54)
Relative importance (separate measures)^b^		
Relative importance of others (3 items)	0.89 (237)	4.98 (±1.23)
Relative importance of self (3 items)	0.92 (239)	5.58 (±1.15)
Relative importance of others (trade-off measure)	NA (237)	4.14 (±1.90)
Relative importance of self (trade-off measure)	NA (237)	5.86 (±1.90)

Note: N varied due to missing values.

^a^ Cronbach’s alpha was calculated for 3-item construct measures and Spearman-Brown Coefficient was calculated for 2-item behavior measures.

^b^ Scored on one to seven scales such as 1 = strongly disagree and 7 = strongly agree.

**TABLE 2:** Frequency of the trade-off measure for the relative importance of others vs self.

Relative importance of others vs self	Frequency (%)
0/10	4 (1.7)
1/9	12 (5.1)
2/8	26 (11.0)
3/7	54 (22.8)
4/6	44 (18.6)
5/5	51 (21.5)
6/4	17 (7.2)
7/3	13 (5.5)
8/2	13 (5.5)
9/1	2 (0.8)
10/0	1 (0.4)
Total N	237 (100)

Note: Total N differed from 239 due to missing values.

The factor analyses showed that the relative importance of self (separate measures) loaded on one factor with self-identity, and the relative importance of others (separate measures) had cross loadings with subjective norm (Table 3). This suggests the separate measures were not good instruments to capture the relative importance. The trade-off measure had low correlations with TPB constructs, which ranged from −0.23 to 0.23. Therefore, the trade-off measure was a better measure than the separate measures, and only the trade-off measure was used in regression analyses.

**TABLE 3:** Exploratory factor analyses for the separate measures of relative importance.

Variables	Factor 1	Factor 2
Factor analysis 1 Relative importance of self (separate measures) and self-identity^a^ ^,^ ^b^		
Relative importance of self item 1	**0.84**	
Relative importance of self item 2	**0.91**	
Relative importance of self item 3	**0.84**	
Self-identity item 1	**0.77**	
Self-identity item 2	**0.84**	
Self-identity item 3	**0.90**	
Factor analysis 2 Relative importance of others (separate measures) and subjective norm ^b^		
Relative importance of others item 1	**0.88**	**0.50**
Relative importance of others item 2	**0.88**	0.40
Relative importance of others item 3	**0.80**	0.38
Subjective norm item 1	**0.64**	0.47
Subjective norm item 2	0.44	**0.77**
Subjective norm item 3	0.43	**0.86**

Note: Principal axis factoring was used to extract factors, oblique rotation was used to rotate factors, and eigenvalue >1 criterion was used to decide the number of factors.

^a^ Only one factor was extracted.

^b^ Scored on one to seven scales such as 1 = strongly disagree and 7 = strongly agree.

Values ≥ 0.5 were bolded.

Both regressions for intention prediction explained 75% of the variance in intention (Table 4). Self-efficacy and attitude were two strong intention predictors. Self-identity was not a significant predictor in the TPB with self-identity, but self-identity weighted by the relative importance of self was a significant predictor in the approach of Norm Balance. In the TPB with self-identity, neither subjective norm nor self-identity was a significant intention predictor across three subgroups (Table 5). In the approach of Norm Balance under TPB, for the subgroup with relative importance of others >4, subjective norm weighted by the relative importance of others was a stronger intention predictor than self-identity weighted by the relative importance of self; and for the subgroup with relative importance of subjective norm <4, self-identity weighted by the relative importance of self was a stronger predictor than subjective norm weighted by the relative importance of others. The regression for behavior prediction explained 30% of the variance in behavior (Table 6). Intention and self-efficacy were the significant predictors of behavior.

**TABLE 4:** Regression comparison for intention prediction between TPB with self-identity vs the approach of Norm Balance.

TPB with Self-identity^a^			
Regression model	Intention of collaborating with the physician (N = 233)		
Adjusted R square	0.75		
Df	4		
F	173.84**		
Independent variables	Standardized beta	Tolerance	VIF
Attitude	0.36**	0.64	1.56
Subjective norm	0.10*	0.64	1.56
Self-efficacy	0.56**	0.76	1.31
Self-identity	0.07	0.72	1.39

The approach of Norm Balance using the trade-off measure			
Regression model	Intention of collaborating with the physician (N = 233)		
Adjusted R square	0.75		
Df	5		
F	175.60**		
Independent variables	Standardized beta	Tolerance	VIF
Attitude	0.37**	0.67	1.49
Subjective norm weighted by the relative importance of others^b^	0.19**	0.42	2.36
Self-efficacy	0.57**	0.76	1.32
Self-identity weighted by the relative importance of self^c^	0.15**	0.37	2.73

Note: N differed from 239 due to missing values.

**Significant at 0.01, *significant at 0.05.

^a^ “TPB with self-identity” is TPB with the addition of self-identity as a framework.

^b^ “Subjective norm weighted by the relative importance of others” is the measurement scores reported for subjective norm times the value reported for “others” in the trade-off measure.

^c^ “Self-identity weighted by the relative importance of self” is the measurement scores reported for self-identify times the value reported for “self” in the trade-off measure.

**TABLE 5:** Regression comparison for subgroups according to relative importance of others using trade-off measure (median as cutoff).

TPB with self-identity^a^			
Regression model	Relative importance of others		
Intention of collaborating with the physician	>4 (N = 94)	= 4 (N = 44)	<4 (N = 95)
Adjusted R square	0.75	0.69	0.74
Df	4	4	4
F	71.07**	25.33**	67.33**
Standardized beta			
Attitude	0.33**	0.63**	0.32**
Subjective norm	**0.08**	**0.07**	**0.12**
Self-efficacy	0.58**	0.21	0.59**
Self-identity	**0.08**	**0.11**	**0.08**

The approach of Norm Balance using the trade-off measure			
Regression model	Relative importance of others		
Intention of collaborating with the physician	>4 (N = 94)	= 4 (N = 44)	<4 (N = 95)
Adjusted R square	0.76	0.69	0.74
Df	4	4	4
F	75.37**	25.34**	67.37**
Standardized beta			
Attitude	0.32**	0.63**	0.34**
Subjective norm weighted by the relative importance of others ^b^	0.20**	**0.07**	**0.10**
Self-efficacy	0.58**	0.21	0.60**
Self-identity weighted by the relative importance of self ^c^	**0.08**	**0.11**	0.13*

Note: Total N of three subgroups (233) differed from 239 due to missing values.

**Significant at 0.01, *significant at 0.05.

^a^ “TPB with self-identity” is TPB with the addition of self-identity as a framework.

^b^ “Subjective norm weighted by the relative importance of others” is the measurement scores reported for subjective norm times the value reported for “others” in the trade-off measure.

^c^ “Self-identity weighted by the relative importance of self” is the measurement scores reported for self-identify times the value reported for “self” in the trade-off measure.

Values for comparison were bolded.

**TABLE 6:** Regression for behavior prediction.

Regression model	Behavior of collaborating with the physician (N = 187)		
Adjusted R square	0.30		
Df	2		
F	40.45**		
Independent variables	Standardized beta	Tolerance	VIF
Intention	0.37**	0.38	2.63
Self-efficacy	0.22*	0.38	2.63

Note: N differed from 188 due to missing values.

**Significant at 0.01, *significant at 0.05.

## Discussion

In a meta-analysis, Armitage and Conner found that the TPB typically accounted for about 39% the variance in intention and about 27% of the variance in behavior (Armitage and Conner, 2001). In this study, 75% of the variance in intention was explained by either the TPB with self-identity or the approach of Norm Balance (Table 4), and 30% of the variance in behavior was explained by the TPB (Table 6). So, the predictive performance for intention was higher than reported in the literature, while the predictive performance for behavior was consistent. As predictability depends on various contextual and methodological factors (Armitage and Conner, 2001), pharmacist-physician collaboration could be a behavior with solid relationships among constructs leading to intention. The 3-month time interval between measuring intention and behavior could affect the predictability of behavior, making the variance in behavior consistent with the literature.

In addition to the trade-off measure, we measured the relative importance of subjective norm and self-identity with the separate measures. The construct validity of these measures was questionable since they had cross factor loadings with the measurement scores (Table 2). Although they separately measured the “importance” of others vs self, the separate measures did not compare which of these two was more important. The trade-off measure is a better instrument than the separate measures because it forces people to rate the relative importance by presenting the two sources together (others and self). Future studies using the approach of Norm Balance could depend on the trade-off measure for the relative importance of others vs self.

Consistent with the study of Liu et al. (Liu et al., 2023), we found that the approach of Norm Balance provided a different view about the significance and coefficients of subjective norm and self-identity. Self-identity was not an intention predictor for collaboration in the TPB with self-identity. But when self-identity was weighted by the relative importance of self, it became a significant predictor in the approach of Norm Balance. We had anticipated that self-identify would predict intention to collaborate. This is because collaboration requires complementary roles of related parties, and specific roles which pharmacists assume in healthcare affect the collaboration (Fagin, 1992; McDonough and Doucette, 2001). For instance, pharmacists could complement physicians with medication therapy expertise, and this specific role as therapeutic experts would be expected by physicians. But self-identity only affected intention in the approach of Norm Balance, which indicates the approach of Norm Balance may be a better model than the TPB with self-identity to examine pharmacist-physician collaboration.

The regression comparisons across subgroups with different relative importance of others also show that the approach of Norm Balance may be a better model. If subjects reported that their important others impacted their behavioral decision more than themselves, we would expect subjective norm to be a stronger predictor than self-identity. In contrast, if subjects reported that themselves impacted their behavioral decision more than their important others, we would expect self-identity to be a stronger intention predictor. Yet, we only observed such results in the approach of Norm Balance. That is, when the relative importance of others was >4, subjective norm weighted by the relative importance of others was a significant predictor, while self-identity weighted by the relative importance of self was not. When the relative importance of others was <4, self-identity weighted by the relative importance of self was a significant predictor, while subjective norm weighted by the relative importance of others was not.

According to the results of the study of Liu et al. (Liu et al., 2023) and this study, the approach of Norm Balance might show advantages over the TPB with self-identity for some behaviors, such as informing relatives about counterfeit medications (Liu et al., 2023) and pharmacist-physician collaboration. Similarities of the two behaviors included 1) another party (i.e., relatives or physicians) was involved in addition to the self, and 2) the behavioral outcome may impact individuals’ important others (i.e., the health of relatives or patients). These similarities distinguished the behaviors from the other five behaviors which involved only the self. Compared with a behavior which involves only the self, individuals may be more attentive to the opinions of others for a behavior which involves and impacts other parties. Individuals may carefully consider their own opinions as well as those others, which could include comparing the relative importance of others vs self. Thus, the approach of Norm Balance became salient for behaviors involving and impacting others.

Self-efficacy and attitude were two strong predictors of intention (Table 4). Pharmacists have extended their practice from dispensing medications to managing medication therapies. With the expertise in therapeutics and skills to provide clinical services, pharmacists are confident to work with physicians to improve medication therapy. Thus, self-efficacy affected intention. Meanwhile, managing medication therapy requires persistence by pharmacists. Having a positive attitude toward collaboration with physicians can support pharmacists’ intention for such collaboration. The average of the attitude measure was 6.20 (Table 1), the highest value among the means of TPB construct measures, which reflects pharmacists indeed had a favorable perception about collaboration.

**FIGURE 1:**
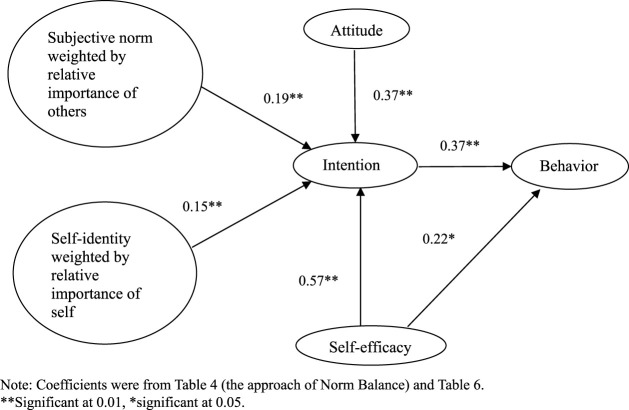
The approach of Norm Balance under TPB with study results.

Intention and self-efficacy were two predictors of behavior (Table 6), which is consistent with how behavior prediction is theorized in the TPB. Previously, we proposed a modified TPB with the approach of Norm Balance (Liu et al., 2023). With the results of this study, now we could summarize the relationship among TPB constructs and the approach of Norm Balance, as shown in Figure 1. Intention prediction is modified through the approach of Norm Balance by weighing subjective norm and self-identity, whereas behavior prediction is the same as the TPB. In this study, we demonstrated the robustness of this modified TPB in both the intention prediction and behavior prediction for pharmacist-physician collaboration to improve medication therapy.

The study had several limitations. Firstly, the sample size was originally planned to be 304 respondents, but the first survey had only 239 respondents, about 80% of the planned sample size. A smaller-than-expected sample size can reduce the statistical power of the study, making it more difficult to detect significant effects, if they exist. Namely, the reduced power increases the risk of Type II errors, in which true effects are not detected. In addition, a smaller sample size may not adequately represent the population, leading to limited generalizability (Liu et al., 2010) and non-response bias (Liu and Doucette, 2011). Another limitation is the time elapse between data collection and now. Since data collection, the extent of pharmacist-physician collaboration has evolved, such as the increased implementation of Collaborative Practice Agreements facilitated by legislative changes (Howell, 2023) and more evidence supporting pharmacists’ role in medication management and patient education (Jaam et al., 2021). Advancements in health information technology have enabled pharmacists to access patient health records and perform medication therapy management more efficiently (Schneider, 2018). In other words, pharmacist-physician collaboration has strengthened, driven by a shared commitment to improving patient care. Nowadays, the relationship between pharmacists’ intention and behavior to collaborate with physicians could be more robust. Nevertheless, because the contextualization of pharmacist-physician collaboration was general in this study, the findings should still apply to examine the relationship between the approach of Norm Balance and pharmacists’ intention to collaborate with physicians. Future studies could explore to include other normative concepts in the approach of Norm Balance. In addition, geographic variation may play a role in the amount and scope of professional interactions. A pharmacist in a small rural town may have stronger relationships with local physicians than a pharmacist in a large metropolitan area. Thus, how geographic variation influences the approach of Norm Balance in pharmacist-physician collaboration may be a future direction of research as well.

## Conclusion

In conclusion, the construct validity of the separate measures was questionable, and the trade-off measure was a better instrument to capture the relative importance of others vs self. In terms of examining pharmacists’ intention to collaborate with physicians, the approach of Norm Balance under the TPB may be a better model than the TPB with self-identity. Additionally, self-efficacy and attitude were two strong predictors of pharmacists’ intention to collaborate with physicians, and intention and self-efficacy were two predictors of collaboration.

## Data Availability

The original contributions presented in the study are included in the article/Supplementary Material, further inquiries can be directed to the corresponding author.
