# Supplementary material for: The approach of Norm Balance in predicting pharmacists’ intention to collaborate with physicians to improve medication therapy
Source: Front Pharmacol. 2024 Sep 16;15:1375529. doi: 10.3389/fphar.2024.1375529 (PMC11456739; doi:10.3389/fphar.2024.1375529)
Supplement: Supplementary file 1 [file DataSheet1.pdf]

Supplementary Material. Measures

TPB constructs and self-identity

Attitude toward behavior
(1) “For me, collaborating with the physician to improve medication therapy is” (“1 = unnecessary” and “7 = necessary”);
(2) “For me, collaborating with the physician to improve medication therapy is” (“1 = negative” and “7 = positive”);
(3) “For me, collaborating with the physician to improve medication therapy is” (“1 = bad” and “7 = good”).

Subjective norm
(1) “People who are important to me would disapprove/approve of my collaborating with the physician to improve medication therapy.” (“1 = disapprove” and “7 = approve”);
(2) “People who are important to me think I should not/should collaborate with the physician to improve medication therapy.” (“1 = should not” and “7 = should”);
(3) “People who are important to me want me to collaborate with the physician to improve medication therapy.” (“1 = strongly disagree” and “7 = strongly agree”).

Self-efficacy
(1) “If you wanted to, how confident are you that you are able to collaborate with the physician in the next three months?” (“1 = not very confident” and “7 = very confident”);
(2) “If you wanted to, to what extent do you see yourself as being capable of collaborating with the physician in the next three months?” (“1 = very incapable” and “7 = very capable”);
(3) “If I wanted to, I believe I am able to collaborate with the physician in the next three months.” (“1 = definitely would not” and “7 = definitely would”).

Self-identity
(1) “I think of myself as a collaborator with the physician to improve medication therapy.” (“1 = strongly disagree” and “7 = strongly agree”);
(2) “I think of myself as someone who is concerned with pharmacist-physician collaboration to improve medication therapy.” (“1 = strongly disagree” and “7 = strongly agree”);
(3) “I think of myself as someone who cares about consequences of pharmacist-physician collaboration to improve medication therapy.” (“1 = strongly disagree” and “7 = strongly agree”).

Intention
(1) “I intend to collaborate with the physician to improve medication therapy in the next three months.” (“1 = definitely do not” and “7 = definitely do”);
(2) “I plan to collaborate with the physician to improve medication therapy in the next three months.” (“1 = definitely do not” and “7 = definitely do”);
(3) “I want to collaborate with the physician to improve medication therapy in the next three months.” (“1 = definitely do not” and “7 = definitely do”).

Behavior [a]

(1) "In the three months since completing the first survey, I collaborated with the physician to improve medication therapy." ("1 = strongly disagree" and "7 = strongly agree");
(2) "In the three months since completing the first survey, how often did you collaborate with the physician to improve medication therapy?" ("1 = never" and "7 = extremely frequently)".

Relative importance of others vs self

The trade-off measure

"Please allocate 10 points between the two sources below to indicate the extent of their impact on your decision to collaborate with the physician to improve medication therapy. Please use whole numbers.

People who are important to you____;   Yourself____"

The separate measures for the relative importance of others

(1) "I highly care about the opinions of people who are important to me on whether to collaborate with the physician to improve medication therapy." ("1 = strongly disagree" and "7 = strongly agree")
(2) "I greatly value the opinions of people who are important to me on whether to collaborate with the physician to improve medication therapy." ("1 = strongly disagree" and "7 = strongly agree")
(3) "The opinions of people who are important to me have strong influence on my decision to collaborate with the physician to improve medication therapy." ("1 = strongly disagree" and "7 = strongly agree")

The separate measures for the relative importance of self

(1) "I highly care about my own opinions on my collaboration with the physician to improve medication therapy." ("1 = strongly disagree" and "7 = strongly agree")
(2) "I greatly value my own opinions on whether to collaborate with the physician to improve medication therapy." ("1 = strongly disagree" and "7 = strongly agree")
(3) "My own opinions have strong influence on my decision to collaborate with the physician to improve medication therapy." ("1 = strongly disagree" and "7 = strongly agree")

Filter question

Are you currently practicing as a pharmacist? (Yes or No) [b]

Note: [a] Behavior was measured in the second survey three months after the first survey.
[b] The filter question was used to exclude pharmacists who were not practicing.
"The physician" in items refers to one physician with whom the pharmacist was interested in working. "The physician" could be one physician with whom the pharmacist is currently working, or one with whom the pharmacist has not worked.
